# Correlação do Fator de Diferenciação de Crescimento-15 com Marcadores Urinários em Pacientes com Insuficiência Cardíaca Crônica

**DOI:** 10.36660/abc.20240153

**Published:** 2025-03-27

**Authors:** Gustavo Rodolfo Moreira, Diane Xavier Ávila, Angelo Michele Di Candia, Victoria Depes Scaramussa, Humberto Villacorta

**Affiliations:** 1 Universidade Federal Fluminense Hospital Universitário Antônio Pedro Niterói RJ Brasil Universidade Federal Fluminense - Hospital Universitário Antônio Pedro, Niterói, RJ – Brasil

**Keywords:** Insuficiência Cardíaca, Biomarcadores, GDF-15, Síndrome Cardio-renal, Testes de Função Renal

## Abstract

**Fundamento:**

Há uma falha em se identificar pacientes com insuficiência cardíaca (IC) com um risco aumentado de hospitalização e morte. O Fator de Diferenciação de Crescimento-15 (GDF-15), um biomarcador associado com inflamação e estresse oxidativo, surge como um candidato associado a doença renal e cardiovascular. Valores baixos de taxa de filtração glomerular estimada (TFGe), Sódio Urinário (NaU), e valores altos da relação albumina/creatinina urinária são marcadores renais já associados com um alto risco de internação hospitalar e mortalidade.

**Objetivos:**

Avaliar a relação entre GDF-15 e marcadores renais em pacientes com IC crônica.

**Métodos:**

Incluímos 87 pacientes consecutivos com IC sintomática e fração de ejeção do ventrículo esquerdo reduzida (FEVE <40%), FEVE levemente reduzida (40-49%) ou FEVE melhorada (50%, mas <50% anteriormente) em um hospital universitário. Comparamos as associações do GDF-15 e a fração N-terminal do pro-peptídeo natriurético do tipo B (NT-proBNP) com marcadores renais, usando testes de correlação e análise de regressão múltipla com nível de significância de 5%.

**Resultados:**

o GDF-15 e o NT-proBNP apresentaram correlações negativas fracas a moderadas com NaU (r=-0,362; p=0,007; r=-0,334; p=0,014, respectivamente) e TFGe (r=-0,385; p=0,0002; r=-0,346; p=0001, respectivamente). GDF-15, idade, hipertensão e NT-proBNP foram independentemente associados com TFGe na análise de regressão múltipla (total, R2=0,30). Somente o GDF-15 se associou independentemente com NaU (R2=0,45).

**Conclusões:**

Nesses pacientes crônicos com IC, o GDF-15 correlaciona-se melhor com marcadores de disfunção renal que o NT-proBNP. Uma vez que o valor prognóstico dos marcadores renais está bem estabelecido, esses achados reforçam o papel prognóstico do GDF-15 na IC crônica.

## Graphical abstract

**Figura Central:**
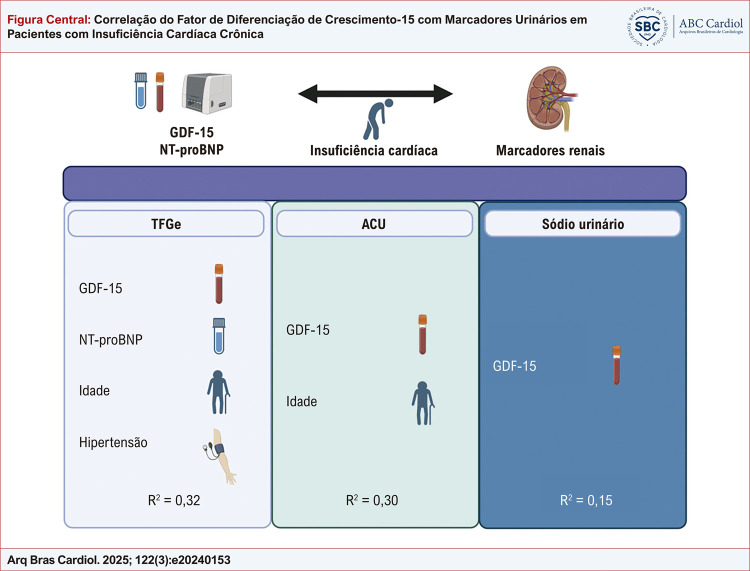
: Correlação do Fator de Diferenciação de Crescimento-15 com Marcadores Urinários em Pacientes com Insuficiência Cardíaca Crônica

## Introdução

A Insuficiência Cardíaca (IC) é um importante problema de saúde pública associado a morbidade, mortalidade e custos elevados. Apesar de tratamentos bem estabelecidos, com benefícios indiscutíveis para pacientes com IC com Fração de Ejeção Ventricular Esquerda (FEVE) reduzida, são necessários melhores preditores de internação hospitalar e mortalidade.^1^

Recentemente, a interface cardiorrenal tem atraído o interesse no contexto da IC devido ao seu papel crucial na congestão e à sua utilidade como ferramenta prognóstica.^2-4^ Uma baixa Taxa de Filtração Glomerular estimada (TFGe), baixos níveis de sódio urinário (NaU) e valores altos da razão Albumina/Creatinina Urinária (ACU) surgiram como bons preditores de internação hospitalar e mortalidade entre os pacientes com IC, independentemente de outros biomarcadores.^2,4-6^

Os biomarcadores exercem um papel essencial no prognóstico da IC, e os peptídeos natriuréticos são considerados padrão-ouro. Contudo, ainda há espaço para melhorias, e a associação entre peptídeos natriuréticos e os biomarcadores que refletem diferentes vias pode aumentar a precisão prognóstica.

O Fator de Diferenciação de Crescimento-15 (GDF-15, do inglês *growth differentiation factor* 15) é um membro da superfamília do fator de transformação do crescimento beta e está associado a estresse oxidativo e inflamação.^7-11^ Embora o GDF-15 não seja um fator específico renal nem cardiovascular, vários estudos encontraram uma associação entre os níveis de GDF-15 e a piora na função renal, fortemente predizendo desfechos piores na IC, principalmente em cenários agudos.^12,13^ Nosso objetivo foi avaliar as associações do GDF-15 e da porção N-terminal do pró-hormônio do peptídeo natriurético do tipo B (NT-proBNP) com a TFGe, NaU e relação ACU em pacientes com IC em tratamento ambulatorial.

## Métodos

Uma amostra de conveniência de pacientes consecutivos com IC crônica em um ambulatório de um hospital universitário terciário foi incluída com base nos seguintes critérios: IC sintomática com FEVE reduzida (FEVE<40%), FEVE levemente reduzida (40-49%) ou FEVE melhorada (50%, mas <50% previamente).

Os pacientes submetidos à terapia de substituição renal e pacientes com história de câncer foram excluídos. O protocolo do estudo foi aprovado pelo comitê de ética do hospital terciário e todos os pacientes assinaram um termo de consentimento para participar.

Este foi um estudo transversal em que foram analisados dados clínicos, biomarcadores sanguíneos e urinários e ecocardiogramas. Utilizamos sexo, Diabetes Mellitus (DM), hipertensão, doença pulmonar obstrutiva crônica (DPOC), fibrilação atrial (FA) e etiologia isquêmica como variáveis categóricas. Idade, pressão arterial sistólica (PAS), pressão arterial diastólica (PAD), frequência cardíaca (FC), FEVE, índice de massa corporal (IMC), TFGe, NT-proBNP, NaU, ACU e GDF-15 foram as variáveis contínuas. A TFGe foi calculada usando a equação do *Chronic Kidney Disease Epidemiology Collaboration* de 2021, baseada na Creatinina Sérica (CrS). Os níveis de NT-proBNP foram determinados usando o Elecsys^®^ proBNP II (Roche Diagnostics GmbH, Mannheim, Alemanha), e os níveis de GDF-15 pelo método de imunoensaio tipo sanduíche com anticorpos monoclonais utilizando o Elecsys^®^ GDF-15 (Roche Diagnostics GmbH, Mannheim, Alemanha).

### Análise estatística

As variáveis categóricas foram expressas em valores absolutos e relativos. As variáveis contínuas foram apresentadas em média e desvio padrão (DP), exceto para variáveis com distribuição não normal, expressas em mediana e Intervalo Interquartil (IIQ). O teste de Kolmogorov-Smirnov foi usado para avaliar a normalidade dos dados. Para correlacionar parâmetros renais com achados clínicos e laboratoriais, um teste de correlação de Pearson foi aplicado para variáveis com distribuição normal e o teste de Spearman para variáveis sem distribuição normal. As variáveis categóricas foram comparadas usando o teste do qui-quadrado ou o teste de McNemar. O teste t de Student para amostras independentes ou o teste de Mann-Whitney (não paramétrico) foi usado para comparar variáveis contínuas. A análise multivariada utilizou regressão linear múltipla para identificar variáveis independentes associadas com NaU e ACU, adaptando essa distribuição para análise paramétrica. A análise estatística foi realizada com o programa SPSS versão 26 (IBM Corporation, Armonk, NY, EUA); diferenças com p < 0,05 foram consideradas estatisticamente significativas.

## Resultados

**Figura 1:**
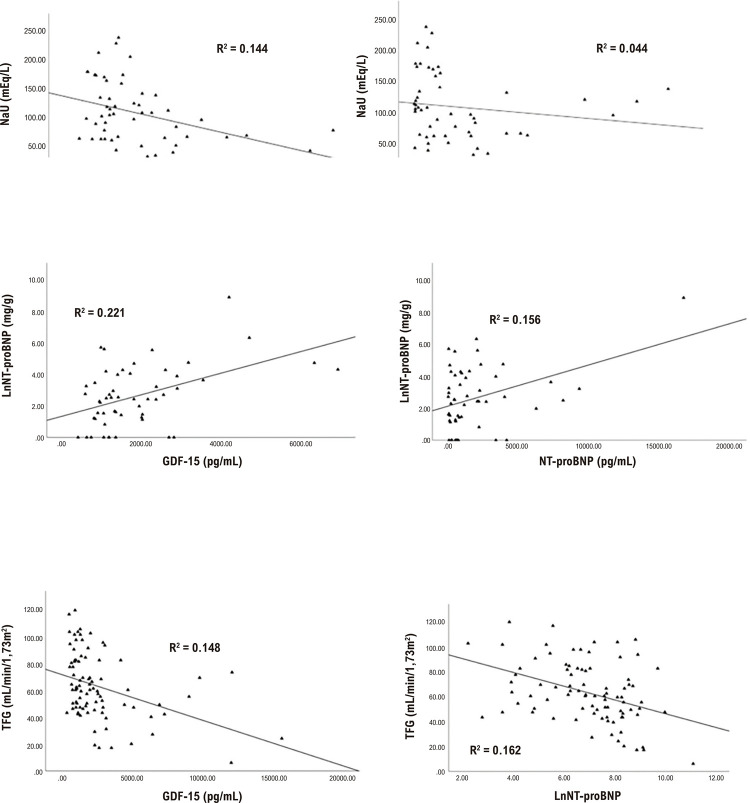
– Correlações do Fator de Diferenciação de Crescimento-15 (GDF-15) e fração-terminal do pró-hormônio do peptídeo natriurético do tipo B (NT-proBNP) com biomarcadores renais; TFGe: Taxa de Filtração Glomerular estimada; LnNT-proBNP: transformação logarítmica natural do NT-proBNP; ACU: relação Albumina/Creatinina Urinária (ACU); NaU: Sódio Urinário.

Oitenta e sete pacientes foram incluídos no estudo: as características basais dos pacientes estão resumidas na Tabela 1. Os valores medianos de GDF-15 e NT-proBNP foram 1398 pg/mL (IIQ 1053–2317 pg/mL) e 729,5 pg/mL (IQR 222,7–2182,5 pg/mL), respectivamente. Os marcadores renais foram igualmente distribuídos entre as comorbidades clínicas, exceto pelo valor mais baixo da TFGe em pacientes com hipertensão (Tabela 2). Os níveis de GDF-15 foram moderadamente e positivamente correlacionados com as concentrações de NT-proBNP (r = 0,538, p < 0,001), creatinina sérica (r = 0,456, p < 0,001), e ureia (r = 0.478, p < 0.001). As associações dos marcadores de função renal com NT-proBNP, GDF-15, e FEVE estão resumidas na Tabela 3. As correlações entre marcadores de função renal e GDF-15 e NT-proBNP estão apresentadas na Figura 1. A análise de regressão linear revelou que um modelo com GDF-15, idade, hipertensão e NT-proBNP foi independentemente associado com TFGe (R^2^ = 0,32), indicando que essas variáveis explicaram 32% da variação na TFGe (Tabela 4). Em um modelo com LogACU como a variável dependente, somente o GDF-15 e a idade mostraram uma correlação significativa (positiva e negativa, respectivamente), com um R2 ajustado de 0,30 (Tabela 5). Somente o GDF-15 foi associado com NaU, e explicou 15% da variação nos seus níveis (Tabela 6). Para esses modelos, a análise de tolerância foi maior que 0,10, e o fator de inflação de variância foi menor que 10 tanto nas variáveis incluídas como nas variáveis excluídas. A estatística de Durbin-Watson variou entre 1,5 e 2,5, sugerindo ausência de autocorrelação. O desvio padrão residual variou entre -3 e 3, e apresentou distribuição normal. O gráfico de dispersão de resíduos e valores ajustados é um padrão aleatório ao redor do zero, sugerindo homoscedasticidade e relações lineares entre variáveis dependentes e independentes.

**Tabela 1:** – Características basais de toda a população (n=87)

Variáveis	Resultados
Idade (anos)	65,9 ± 12,1
Sexo masculino	53 (60,9%)
Hipertensão	73 (83,9%)
Diabetes Mellitus	29 (33,3%)
Etiologia isquêmica	30 (34,5%)
DPOC	19 (21,8%)
FA	31 (35,6%)
DRC	41 (47,1%)
Diuréticos	35 (48,6%)
Furosemida +HCTZ	8 (11,2%)
Bloqueador do SRAA	65 (90,1%)
BB	58 (81,7%)
PAS (mmHg)	126,9 ± 19,8
PAD (mmHg)	78,0 ± 10,9
FC (bpm)	76,1 ± 13,0
IMC (Kg/m^2^)	27,4 ± 4,7
Dose de furosemida (mg/dia)	66,0 ± 53,4
GDF-15 (pg/mL)	1808 (1059-2894)
NT-proBNP (pg/mL)	1343 (443-4010)
FEVE (%)	37,5 ± 11,8
FEVE < 40%	46 (52,9%)
FEVE 40-49%	36 (41,4%)
NaU (mEq/L)	100 (62,5-138)
ACU (mg/g)	12,1 (3,48-56,0)
eGFR (mL/min/1,73m^2^)	62,5±24,2

FA: Fibrilação Atrial; BB: Betabloqueador; IMC: Índice de Massa Corporal; DRC: Doença Renal Crônica; DPOC: Doença Pulmonar Obstrutiva Crônica; PAD: Pressão Arterial Diastólica; DM: Diabetes Mellitus; TFGe: Taxa de Filtração Glomerular estimada; GDF-15: Fator de Diferenciação de Crescimento-15; HCTZ: Hidroclorotiazida; FC: Frequência Cardíaca; FEVE: Fração de Ejeção do Ventrículo Esquerdo; NT-proBNP: fração N-Terminal do pró-hormônio do peptídeo natriurético do tipo B; SRAA: Sistema Renina Angiotensina Aldosterona; PAS: Pressão Arterial Sistólica; ACU: relação Albumina/Creatinina Urinária; NaU: Sódio Urinário

**Tabela 2:** – Marcadores da função renal segundo o sexo e comorbidades

Variável	NaU		ACU		TFGe	
	Mediana (IIQ)	Valor p	Mediana (IIQ)	Valor p	Média ± DP	Valor p
**Sexo**						
Masculino	104 (65-161)	0,36	12,5 (4,0-57,0)	0,64	59,3 ± 23,9	0,12
Feminino	94 (61-121)		11,1 (2,0-52,0)		67,5 ± 24,1	
**Hipertensão**						
Sim	102 (62-149)	0,82	12,5 (3,0-62,0)	0,84	59,7 ± 21,3	0,07
Não	98 (94-137)		11,3 (4,0-33,0)		77,2 ± 32,7	
**DM**						
Sim	76 (60-119)	0,21	15,9 (2,0-102,0)	0,48	64,8 ± 24,6	0,53
Não	106 (64-160)		11,3 (3,0-35,0)		61,3 ± 24,1	
**Etiologia**						
Sim	105 (61-169)	0,82	34,3 (1,0-105,0)	0,55	58,6 ± 19,2	0,27
Não	96 (64-134)		11,3 (4,0-29,0)		64,6 ± 26,3	
**DPOC**						
Sim	87 (59-210)	0,76	4,7 (1,0-32,0)	0,19	70,0 ± 21,7	0,12
Não	101 (63-133)		13,6 (4,0-68,0)		60,4 ± 24,5	
**FA**						
Sim	107 (65-145)	0,65	11,1 (4,0-32,0)	0,93	57,9 ± 24,3	0,18
Não	100 (61-139)		12,1 (3,0-66,0)		65,1 ± 23,9	

TFGe: Taxa de Filtração Glomerular estimada; ACU: relação Albumina/Creatinina Urinária; NaU: Sódio Urinário; DPOC: Doença Pulmonar Obstrutiva Crônica; FA: Fibrilação Atrial

**Tabela 3:** – Correlações de marcadores renais com parâmetros clínicos, N-terminal do pró-hormônio do peptídeo natriurético do tipo B (NT-proBNP) e Fração de Ejeção do Ventrículo Esquerdo (FEVE)

Variável	NaU		ACU		TFGe	
	r	Valor p	r	Valor p	r	Valor p
Idade (a)	0,111	0,43	-0,159	0,26	-0,288*	0,006
PAS (mmHg)	0,178	0,22	0,089	0,54	-0,145	0,19
PAD (mmHg)	0,183	0,20	0,235	0,10	-0,124	0,26
FC (bpm)	-0,060	0,68	0,150	0,30	0,011	0,92
IMC (Kg/m^2^)	0,054	0,70	-0,127	0,37	0,064	0,56
GDF-15 pg/mL	-0,362*	0,007	0,400**	0,003	-0,385*	0,0002
NT-proBNP (pg/mL)	-0,334*	0,014	0,189	0,17	-0,346*	0,001
FEVE (%)	0,269	0,051	-0,073	0,60	0,209	0,052

r: Coeficiente de correlação de Spearman para Sódio Urinário (NaU) e relação Albumina/Creatinina Urinária (ACU), e coeficiente de Pearson para Taxa de Filtração Glomerular estimada (TFGe); números em negrito indicam significância estatística. * correlação fraca; ** correlação moderada. IMC: Índice de Massa Corporal; PAS: Pressão Arterial Sistólica; PAD: Pressão Arterial Diastólica; FC: Frequência Cardíaca; FEVE: Fração de Ejeção Ventricular Esquerda; NT-proBNP: fração N-Terminal do pró-hormônio do peptídeo natriurético do tipo B.

**Tabela 4:** – Regressão linear múltipla para identificar preditores independentes da variação da Taxa de Filtração Glomerular estimada (TFGe) (R**2**=0,32)

Variável	Coeficiente β	Erro padrão β	Valor p	R	R2 ajustado
Constante	121,14	12,28	< 0,0001		
GDF-15	-0,0022	0,0009	0,016	0,39	0,14
Idade (a)	-0,5631	0,1846	0,003	0,50	0,23
Hipertensão	-14,803	6,1726	0,018	0,56	0,28
NT-proBNP	-0,0007	0,0003	0,022	0,59	0,32

Método de seleção por stepwise foward. R: coeficiente de correlação acumulada. R2 ajustado: coeficiente de determinação ajustado; TFGe: Taxa de Filtração Glomerular estimada; GDF-15: Fator de Diferenciação de Crescimento-15; NT-proBNP: fração N-Terminal do pró-hormônio do peptídeo natriurético do tipo B

**Tabela 5:** – Regressão linear múltipla para identificar preditores independentes da variação no logACU (razão Albumina/Creatinina Urinária) (R2=0,30)

Variáveis	Coeficiente β	Erro padrão β	Valor p	R	R2 ajustado
Constante	4,4908	1,2118	0,0005		
GDF-15	0,0007	0,0002	0,0001	0,47	0,21
Idade (anos)	-0,0505	0,0184	0,008	0,57	0,30

Método de seleção por stepwise. R: coeficiente de correlação acumulada. R2 ajustado: coeficiente ajustado acumulado de determinação; TFGe: Taxa de Filtração Glomerular estimada; GDF-15: Fator de Diferenciação de Crescimento-15; LogACU: transformação logarítmica da relação Albumina/Creatinina Urinária

**Tabela 6:** – Regressão linear múltipla para identificar preditores independentes da variação no logNaU (Sódio Urinário)

Variável	Coeficiente β	Erro padrão β	Valor p	R	R2 ajustado
Constante	4,8475	0,1282	< 0,0001		
GDF-15	-0,0002	0,0001	0,003	0,40	0,15

Método de seleção por stepwise. R: coeficiente de correlação acumulada. R2 ajustado: coeficiente ajustado acumulado de determinação; TFGe: Taxa de Filtração Glomerular estimada; GDF-15: Fator de Diferenciação de Crescimento-15; LogNaU: transformação logarítmica do sódio urinário

## Discussão

O presente estudo demonstrou que o GDF-15, um biomarcador de inflamação e estresse oxidativo, associou-se independentemente com níveis mais baixos de NaU e TFGe e valores mais altos de ACU em pacientes com IC crônica. Entre as variáveis habitualmente associadas com IC, somente GDF-15 associou-se de maneira independente com NaU.

A prevalência de Doença Renal Crônica (DRC) na população de nosso estudo foi de 47%, similar à maioria dos estudos com pacientes com IC, variando entre 40% a 60%, e bem mais alta que a observada na população geral (6% a 12%). Segundo uma metanálise, a presença de DRC em pacientes com IC aumenta o risco de mortalidade em 2,34 IC 95% 2,20–2,50; p < 0,0001).^14-16^

O conceito de marcadores renais supera a simples medida da creatinina sérica. Baixa TFG, albuminúria e NaU são marcadores de alterações hemodinâmicas, disfunção endotelial e função tubular, respectivamente.^2,5,17^ A albuminúria é uma doença endotelial e um marcador precoce de doença cardiovascular. Ainda, os níveis de NaU estão inversamente relacionados às concentrações de catecolaminas, renina, e angiotensina II; assim, os níveis de NaU podem refletir hiperativação neuro-humoral e são considerados marcadores de resistência diurética.^18-20^

Os mecanismos envolvidos na resistência diurética parece ser multifatorial e incluir ativação do sistema renina-angiotensina-aldosterona e do sistema nervoso simpático, remodelamento do néfron, alterações funcionais renais prévias, e ruptura da farmacocinética e dinâmica diurética.^21^

O GDF-15 tem sido associado com desfechos renais e cardiovasculares ruins durante a internação em pacientes com IC e naqueles submetidos a procedimentos cirúrgicos e intervencionistas.^22-25^ O GDF-15 também é um bom preditor de eventos em pacientes com IC crônica. Além disso, altos níveis de GDF-15 parecem estar associados com menor uso de terapia modificadora de desfechos e maior uso de diuréticos.^25,27^ Algumas características adicionais tornam o GDF-15 um excelente marcador prognóstico. Primeiramente, sua expressão é fraca em indivíduos sadios, exceto por um leve aumento com a idade. Em segundo, o GDF-15 é um biomarcador sistêmico secretado por uma grande variedade de células e, por isso, pode refletir as repercussões sistêmicas da IC.

O racional para o uso do GDF-15 no contexto cardiorrenal baseia-se no fato de que, mesmo nos pacientes assintomáticos sem níveis elevados de NT-proBNP ou congestão clara, a perfusão tecidual pode ser inadequada. Identificar má perfusão tecidual, levando à ativação neuro-hormonal, é função inerente aos rins e o dano tecidual renal é um estímulo para a síntese do GDF-15. Portanto, o GDF-15, como um marcador de sofrimento tecidual, pode ser um marcador do risco de uma real piora na função renal em pacientes com IC.

Em nosso estudo, o GDF-15, mas não o NT-proBNP, correlacionou-se independentemente com marcadores de lesão renal (isto é, ACU e NaU). Por outro lado, NT-proBNP foi um melhor preditor independente da função renal que o GDF-15, conforme avaliado por sua correlação com a TFGe. Os níveis de NT-proBNP são afetados por congestão, o qual está fortemente associado com função renal. Outros estudos sugeriram que os níveis de GDF-15 podem refletir lesão renal. Nair et al.^9^ demonstraram uma correlação positiva robusta da expressão do RNA mensageiro para GDF-15 nos rins com os níveis de GDF-15 sérico, e uma correlação negativa com TFGe.^9^ Em outro estudo, o GDF-15 foi melhor que o NT-proBNP em predizer a progressão de doença renal, definida como doença renal terminal ou uma redução sustentada de 50% na TFGe, em uma coorte prospectiva de pacientes com DRC moderada (TFGe 20- 70 mL/min/1,73 m^2^).^7^ Em conjunto, os resultados desses estudos sugerem que o GDF-15 pode ser um preditor precoce de DRC em pacientes com IC.

O presente estudo teve algumas limitações. Primeiro, este foi um estudo unicêntrico, com um tamanho amostral pequeno, e assim sujeito a erros do tipo 1 e do tipo 2. Segundo, medimos somente os níveis de GDF-15 no basal e não sabemos se medidas seriadas teriam alterado as associações ao longo do tempo. Apesar dessas limitações, nossos achados foram consistentes.

O GDF-15 parece um biomarcador valioso de IC e DRC, e pode ser usado como um preditor de desfechos cardiorrenais.^28^ Sua correlação com marcadores renais sugere que ele pode ser usado para identificar pacientes e com alto risco de piora na função renal, apoiando, assim, o escalonamento da terapia para prevenir tais complicações. Por exemplo, os pacientes com níveis elevados do GDF-15 podem se beneficiar significativamente de drogas com perfis renais mais seguros, tais como o sacubitril/valsartana (que são superiores aos inibidores de enzima conversora de angiotensina e bloqueadores de receptor de angiotensina em termos de desfechos renais^29^) e inibidores do cotransportador de sódio-glicose.^30-32^

## Conclusão

O GDF-15 é um biomarcador emergente associado à lesão renal e cardiovascular e se correlacionou com níveis piores de biomarcadores renais, incluindo NaU, TFGe e ACU. Portanto, o GDF-15 pode ser um marcador precoce de disfunção renal em pacientes com IC, identificando os pacientes com risco aumentado de progressão para doença renal.
