# Supplementary material for: Correlação do Fator de Diferenciação de Crescimento-15 com Marcadores Urinários em Pacientes com Insuficiência Cardíaca Crônica
Source: Arq Bras Cardiol. 2025 Mar 27;122(3):e20240153. [Article in Portuguese] doi: 10.36660/abc.20240153 (PMC12058138; doi:10.36660/abc.20240153)
Supplement: Translation

# Translation: Growth Differentiation Factor 15 is Correlated With Urinary Markers in Patients with Chronic Heart Failure

**DOI:** 10.36660/abc.20240153i

**Authors:** Gustavo Rodolfo Moreira, Diane Xavier Ávila, Angelo Michele Di Candia, Victoria Depes Scaramussa, Humberto Villacorta

**Affiliations:** 1 Brazil Universidade Federal Fluminense - Hospital Universitário Antônio Pedro, Niterói, RJ – Brazil

**Keywords:** Heart Failure, Biomarkers, GDF-15, Cardio-renal Syndrome, Kidney Function Tests

## Abstract

**Background:**

There is a lack in identifying heart failure (HF) patients with an increased risk of hospitalization and death. Growth differentiation factor 15 (GDF-15), a biomarker associated with inflammation and oxidative stress, emerges as a candidate associated with cardiovascular and renal disease. The low estimated glomerular filter rate (eGFR), low urinary sodium (UNa), and the high ratio of albumin to creatinine are renal markers already associated with a high risk of hospitalization and mortality.

**Objective:**

To evaluate the relationship between GDF-15 and renal markers in patients with chronic HF.

**Method:**

We enrolled 87 consecutive patients with symptomatic HF with reduced left ventricular ejection fraction (LVEF < 40%), mildly reduced LVEF (40–49%) or an improved LVEF (50% but previously < 50%) in a university hospital. We compared the associations of GDF-15 and NT-proBNP with renal markers using correlation tests and multiple regression analysis at the significance level of 5%.

**Results:**

GDF-15 and NT-proBNP had weak to moderate negative correlations with UNa (r=-0.362, p=0.007; r=-0.334, p=0.014, respectively) and eGFR (r=-0.385, p=0.0002; r=-0.346, p=0.001, respectively). GDF-15, age, hypertension and NT-proBNP were independently associated with eGFR in multiple regression analysis (overall R2=0.32). GDF-15 (positive) and age (negative) were independently associated with UAC (overall, R2=0.30). Only GDF-15 was independently associated with UNa (R2=0.45).

**Conclusions:**

In these chronic patients with HF, GDF-15 is better correlated with markers of renal dysfunction than NT-proBNP. Since the prognostic value of renal markers is well established, these findings reinforce the prognostic role of GDF-15 in chronic HF.

## Introduction

Heart failure (HF) is a significant public health problem associated with high morbidity, mortality, and cost. Despite well-established medical treatments with undeniable benefits for patients with HF and reduced left ventricular ejection fraction (LVEF), better predictors of hospitalization and mortality are needed.^1^

Recently, the cardiorenal interface has attracted interest in the context of HF due to its crucial role in congestion and its utility as a prognostic tool.^2-4^ A low estimated glomerular filtration rate (eGFR), low urinary sodium level (UNa) and high urinary albumin-to-creatinine ratio (UAC) have emerged as good predictors of hospitalization and mortality among patients with HF, independent of other biomarkers.^2,4,6^

Biomarkers play an essential role in the prognosis of HF, and natriuretic peptides are the gold standard metrics. However, there is still room for improvement, and the association between natriuretic peptides and biomarkers that reflect different pathways can increase prognostic accuracy.

Growth differentiation factor 15 (GDF-15) is a member of the transforming growth factor beta superfamily and is associated with oxidative stress and inflammation.^7-11^ Although GDF-15 is neither renal nor cardiovascular-specific, several studies have found an association between GDF-15 levels and worsening renal function, strongly predicting poor outcomes in HF, especially in acute scenarios.^12,13^ We sought to evaluate the associations of GDF-15 and N-terminal pro-B-type natriuretic peptide (NT-proBNP) with eGFR, UNa, and UAC in outpatient patients with HF.

## Methods

A convenience sample of consecutive patients with chronic HF in an outpatient clinic of a tertiary university hospital was included based on the following criteria: symptomatic HF with reduced LVEF (LVEF < 40%), mildly reduced LVEF (40–49%) or improved LVEF (50% but previously < 50%).

Patients who underwent renal replacement therapy and/or those with a history of cancer were excluded. The study protocol was approved by the ethics committee of the tertiary hospital and all patients provided written informed consent to participate.

This was a cross-sectional study; clinical data, blood and urinary biomarkers, and echocardiograms were analyzed. We used sex, diabetes mellitus (DM), hypertension, chronic obstructive pulmonary disease (COPD), atrial fibrillation (AF), and ischemic etiology as categorical variables. Age, systolic blood pressure (SPB), diastolic blood pressure (DBP), heart rate (HR), LVEF, body mass index (BMI), eGFR, NT-proBNP, and GDF15 were the continuous variables. The eGFR was calculated using the 2021 Chronic Kidney Disease Epidemiology Collaboration equation based on serum creatinine (SCr). The levels of NT-proBNP were determined using Elecsys^®^ proBNP II (Roche Diagnostics GmbH, Mannheim, Germany), and those of GDF-15 by the sandwich immunoassay method with monoclonal antibodies using the Elecsys^®^ GDF-15 (Roche Diagnostics GmbH, Mannheim, Germany).

### Statistical analysis

Categorical variables are expressed as absolute and relative values. Continuous variables are presented as mean and standard deviation (SD), except for non-normal variables, expressed as the median and interquartile range (IQR). The Kolmogorov–Smirnov test was used to evaluate data normality. To correlate renal parameters with clinical and laboratory findings, a Pearson correlation test was performed for normally distributed variables and a Spearman test for nonnormally distributed variables. Categorical variables were compared using the chi-square test or McNemar’s test. Student’s *t test* for independent samples or the Mann–Whitney U test (nonparametric) was used to compare continuous variables. Multivariate analysis used multiple linear regression to identify independent variables associated with UNa, UAC, and eGFR. Correlation coefficients (R) and determination coefficients (adjusted R2) were calculated. A logarithmic transformation was applied to UNa and UAC, adapting this distribution for parametric analysis. Statistical analysis was performed with SPSS version 26 (IBM Corporation, Armonk, NY, USA); differences with p < 0.05 were considered statistically significant.

## Results

Eighty-seven patients were enrolled in the study; baseline characteristics of the study cohort are summarized in Table 1. The median levels of GDF-15 and NT-proBNP were 1,398 pg/mL (IQR 1,053–2,317 pg/mL) and 729.5 pg/mL (IQR 222.7–2182.5 pg/mL), respectively. Renal markers were equally distributed among clinical comorbidities, except for a lower eGFR in patients with hypertension (Table 2). GDF-15 levels were moderately and positively correlated with NT-proBNP (r = 0.538, p < 0.001), serum creatinine (r = 0.456, p < 0.001), and urea levels (r = 0.478, p < 0.001). The associations of renal function markers with NT-proBNP, GDF-15, and LVEF are summarized in Table 3. Correlations of renal function markers with GDF-15 and NT-proBNP are shown in Figure 1. Multiple linear regression analysis revealed that a model with GDF-15 level, age, hypertension, and NT-proBNP level was independently associated with eGFR (R^2^ = 0.32), indicating that these variables explained 32% of the variation in eGFR (Table 4). In a model with LogUAC as the dependent variable, only GDF-15 (positive) and age (negative) were significantly correlated, with an overall adjusted R^2^ of 0.30 (Table 5). Only GDF-15 was associated with UNa, and explained 15% of the variation in its levels (Table 6). For these models, the tolerance statistics were greater than 0,10 and the variance inflation factor was smaller than 10 in both the included and excluded variables. Durbin-Watson statistics ranged from 1.5 to 2.5, suggesting the absence of autocorrelation. Residual standard deviation ranged from -3 to 3 and was normally distributed. The dispersion plot of residuals and fitted values is a random pattern around zero, suggesting homoscedasticity and linear relationships between independent and dependent variables.

**Figure 1:** – Correlations of GDF-5 and NT-proBNP with renal markers. eGFR: estimated Glomerular Flow Rate; GDF-15: Growth Differentiation Factor-15; LnNT-proBNP: Natural Logarithmic transformation of N-terminal pro-B-type natriuretic peptide; LnUAC: Natural Logarithmic transformation of Urinary Albumin to creatinine ratio; UAC: Unrinary albumin to cretainie ratio; NT-proBNP: N-terminal pro-B-type natriuretic peptide; R: determination coefficient; Una: Urinary sodium.

## Discussion

The present study found that GDF-15, a biomarker of inflammation and oxidative stress, was independently associated with lower UNa levels and eGFR and higher UAC ratio in patients with chronic HF. Among the habitual variables associated with HF, only the level of GDF-15 was independently associated with the level of UNa.

The prevalence of chronic kidney disease (CKD) in our study cohort was 47%, which is similar to most studies with HF patients, ranging from 40% to 60%, and much higher than that observed in the general population (6% to 12%). According to a meta-analysis, the presence of CKD in patients with HF increases the risk for mortality by 2.34 (95% confidence interval 2.20–2.50; p < 0.0001).^14-16^

The concept of renal biomarkers overcomes the simple measure of SCr. Reduced eGFR, albuminuria and UNa are markers of hemodynamic abnormalities, endothelial dysfunction, and tubular function, respectively.^2,5,17^ Albuminuria is an endothelial disease and an early marker of cardiovascular disease. Furthermore, UNa levels are inversely related to catecholamine, renin, and angiotensin II; therefore, UNa levels may reflect neurohumoral hyperactivation and are considered markers of diuretic resistance.^18-20^

Mechanisms involved in diuretic resistance appear to be multifactorial and include activation of the renin-angiotensin-aldosterone system and sympathetic nervous system, remodeling of the nephron, previous renal function alterations, and disruption of diuretic pharmacokinetics and dynamics.^21^

GDF-15 has been associated with poor renal and cardiovascular in-hospital outcomes in patients with acute HF and those undergoing surgical and interventional procedures.^22-25^ Similarly, GDF-15 is a good predictor of events in patients with chronic HF.^25,26^ In addition, high levels of GDF-15 appear to be associated with lower use of guideline medical-directed therapy (GDMT) and higher use of diuretics.^26,27^ These additional characteristics make GDF-15 an excellent prognostic marker. First, it is weakly expressed in healthy individuals, except for a slight increase with age. Second, GDF-15 is a systemic biomarker secreted by a wide variety of cells and, as such, may reflect the systemic repercussions of HF.

The rationale for the use of GDF-15 in the cardiorenal context is because even in asymptomatic patients without elevated levels of NT-proBNP or obvious congestion, tissue perfusion may be inadequate. Identifying poor tissue perfusion, leading to neurohormonal activation, is the only role of the kidneys. Renal tissue damage is a stimulus for the synthesis of GDF-15. Therefore, GDF-15, as a marker of tissue suffering, may be a marker of the risk of really worsening renal function in patients with HF.

In our study, GDF-15, but not NT-proBNP, was independently correlated with markers of renal injury (i.e., UAC and UNa). On the contrary, NT-proBNP was a better independent predictor of renal function than GDF-15, as assessed by its correlation with eGFR. NT-proBNP levels are affected by congestion, which is strongly associated with renal function. Other studies have suggested that GDF-15 levels may reflect kidney injury. Nair et al.^9^ demonstrated a robust positive correlation of GDF-15 messenger RNA expression in the kidneys with serum GDF-15 levels, and a negative correlation with eGFR.^9^ In another study, GDF-15 was better than NT-proBNP in predicting the progression of kidney disease, defined as end-stage renal disease or a sustained 50% decrease in eGFR, in a prospective cohort of patients with mild to moderate CKD (eGFR 20–70 mL/min/1.73 m^2^).^7^ Collectively, the results of these studies suggest that GDF-15 may be an early predictor of CKD in patients with HF.

The present study had some limitations. First, it had a single-center design and a small sample size, subject to type 1 and type 2 errors. Second, we only measured GDF-15 levels at baseline and are unsure whether serial measurements would have altered the associations over time. Despite these limitations, our findings were consistent.

GDF-15 appears to be a valuable biomarker of HF and CKD and can be used to predict cardiorenal outcomes.^28^ Its correlation with renal markers suggests that it may be used to identify patients at high risk for worsening renal function, thus supporting the escalation of therapy to prevent such complications. For example, patients with elevated levels of GDF-15 may benefit significantly from the use of drugs with safer renal profiles, such as sacubitril/valsartan (which are superior to angiotensin-converting enzyme inhibitors and angiotensin receptor blockers in terms of renal outcomes^29^) and sodium-glucose cotransporter inhibitors.^30-32^

## Conclusion

GDF-15 is an emerging biomarker associated with kidney and cardiovascular damage and was correlated with worse renal biomarkers, including UNa, eGFR, and UAC. Therefore, GDF-15 may be an early marker of renal disfunction in patients with HF, identifying patients at increased risk for progression of kidney disease.

**Table 1:** – Baseline Characteristics of the entire population (n=87)

Variables	Results
Age (years)	65.9 ± 12.1
Male sex	53 (60.9%)
Hypertension	73 (83.9%)
Diabetes Mellitus	29 (33.3%)
Ischemic etiology	30 (34.5%)
COPD	19 (21.8%)
AF	31 (35.6%)
CKD	41 (47.1%)
Diuretic use	35 (48,6%)
Furosemide +HCTZ	8 (11.2%)
RAAS blocker	65 (90.1%)
BB	58 (81.7%)
SPB (mm Hg)	126.9 ± 19.8
DBP (mm Hg)	78.0 ± 10.9
HR (bpm)	76.1 ± 13.0
BMI (kg/m^2^)	27.4 ± 4.7
Furosemide dose (mg/day)	66.0 ± 53.4
GDF-15 (pg/mL)	1808 (1059-2894)
NT-proBNP (pg/mL)	1343 (443-4010)
LVEF (%)	37.5 ± 11.8
LVEF < 40%	46 (52.9%)
LVEF 40-49%	36 (41.4%)
UNa (mEq/L)	100 (62.5-138)
UAC (mg/g)	12.1 (3.48-56.0)
eGFR (ml/min/1.73m^2^)	62.5±24.2

AF: atrial fibrillation; BB: betablocker; BMI: body mass index; CKD: chronic kidney disease; COPD: chronic obstructive pulmonary disease; DBP: diastolic blood pressure; DM: diabetes mellitus; eGFR: estimated glomerular filtration rate; GDF-15: growth differentiation factor-15; HCTZ: hydrochrolothiazide; HR: heart rate; LVEF: left ventricle ejection fraction; NT-proBNP: N-terminal pro-B-type natriuretic peptide; RAAS: renin-angiotensin-aldosterone system; SBP: systolic blood pressure; UAC: urinary albumin to creatinine ratio; UNa: urinary sodium

**Table 2:** – Values of markers of renal function according to sex and comorbidities

Variable	UNa		UAC		eGFR	
	Median (IQR)	p-value	Median (IQR)	p-value	Mean ± SD	p-value
**Sex**						
male	104 (65-161)	0.36	12.5 (4.0-57.0)	0.64	59.3 ± 23.9	0.12
female	94 (61-121)		11.1 (2.0-52.0)		67.5 ± 24.1	
**Hypertension**						
Yes	102 (62-149)	0.82	12.5 (3.0-62.0)	0.84	59.7 ± 21.3	0.07
no	98 (94-137)		11.3 (4.0-33.0)		77.2 ± 32.7	
**DM**						
yes	76 (60-119)	0.21	15.9 (2.0-102.0)	0.48	64.8 ± 24.6	0.53
no	106 (64-160)		11.3 (3.0-35.0)		61.3 ± 24.1	
**Ischemic etiology**						
yes	105 (61-169)	0.82	34.3 (1.0-105.0)	0.55	58.6 ± 19.2	0.27
no	96 (64-134)		11.3 (4.0-29.0)		64.6 ± 26.3	
**COPD**						
yes	87 (59-210)	0.76	4.7 (1.0-32.0)	0.19	70.0 ± 21.7	0.12
no	101 (63-133)		13.6 (4.0-68.0)		60.4 ± 24.5	
**AF**						
yes	107 (65-145)	0.65	11.1 (4.0-32.0)	0.93	57.9 ± 24.3	0.18
no	100 (61-139)		12.1 (3.0-66.0)		65.1 ± 23.9	

UAC: urinary albumin to creatinine ratio; UNa: urinary sodium; eGFR: estimated glomerular filtration rate; COPD: chronic obstructive pulmonary disease

**Table 3:** – Correlations of renal markers with clinical parameters, left ventricle ejection fraction (LVEF); and N-terminal pro-B-type natriuretic peptide (NT-proBNP)

Variable	UNa		UAC		eGFR	
	r	p-value	r	p-value	r	p-value
Age (y)	0.111	0.43	-0.159	0.26	-0.288*	0.006
SBP (mmHg)	0.178	0.22	0.089	0.54	-0.145	0.19
DBP (mmHg)	0.183	0.20	0.235	0.10	-0.124	0.26
HR (bpm)	-0.060	0.68	0.150	0.30	0.011	0.92
BMI (Kg/m^2^)	0.054	0.70	-0.127	0.37	0.064	0.56
GDF-15 pd/mL	-0.362*	0.007	0.400**	0.003	-0.385*	0.0002
NT-proBNP (pg/mL)	-0.334*	0.014	0.189	0.17	-0.346*	0.001
LVEF (%)	0.269	0.051	-0.073	0.60	0.209	0.052

r: Spearman correlation coefficient for UNa and UAC and Pearson coefficient for eGFR. Bold marks significance. * Weak correlation; ** Moderate correlation. BMI: body mass index; DBP: diastolic blood pressure; eGFR: estimated glomerular filtration rate; HR: heart rate; LVEF: left ventricular ejection fraction; NT-proBNP: N-terminal pro-B-type natriuretic peptide; UAC: urinary albumin to creatinine ratio; UNa: urinary sodium

**Table 4:** – Multiple linear regression to identify independent predictors of estimated glomerular filtration rate (eGFR) variation (overall R^**2**^=0.32)

Variable	Coefficient β	Std Error β	p-value	R	Adjusted R2
Constant	121.14	12.28	< 0,0001		
GDF-15	-0.0022	0.0009	0.016	0.39	0.14
Age (y)	-0.5631	0.1846	0.003	0.50	0.23
Hypertension	-14.803	6.1726	0.018	0.56	0.28
NT-proBNP	-0.0007	0.0003	0.022	0.59	0.32

Selection method by stepwise forward. R: accumulated correlation coefficient. Adjusted R2: accumulated adjusted coefficient of determination. eGFR: estimated glomerular filtration rate; GDF-15: growth differentiation factor-15; NT-proBNP: N-terminal pro-B-type natriuretic peptide.

**Table 5:** – Multiple linear regression to identify independent predictors of logUAC variation (overall R^**2**^=0.30)

Variables	Coefficient β	Std Error β	p-value	R	Adjusted R2
Constant	4.4908	1.2118	0.0005		
GDF-15	0.0007	0.0002	0.0001	0.47	0.21
Age (y)	-0.0505	0.0184	0.008	0.57	0.30

Selection method by stepwise forward. R: accumulated correlation coefficient. Adjusted R2: accumulated adjusted coefficient of determination. GDF-15: Growth differentiation factor-15; LogUAC: Logarithmic transformation of Urinary Albumin to Creatinine ratio.

**Table 6:** – Multiple linear regression to identify independent predictors of logUNa variation

Variable	Coefficient β	Std Error β	p-value	R	Adjusted R2
Constant	4.8475	0.1282	< 0,0001		
GDF-15	-0.0002	0.0001	0.003	0.40	0.15

Selection method by stepwise forward. R: accumulated correlation coefficient. Adjusted R^2^: accumulated adjusted coefficient of determination. GDF-15: Growth differentiation factor-15; LogUNa: Logarithmic transformation of Urinary Sodium
